# Supplementary material for: Cholesterol bound Plasmodium falciparum co-chaperone ‘PFA0660w’ complexes with major virulence factor ‘PfEMP1’ via chaperone ‘PfHsp70-x’
Source: Sci Rep. 2019 Feb 25;9:2664. doi: 10.1038/s41598-019-39217-y (PMC6389991; doi:10.1038/s41598-019-39217-y)
Supplement: Supplementary file 1 — Supplementary information [file 41598_2019_39217_MOESM1_ESM.pdf]

Supplementary Information

Cholesterol bound *Plasmodium falciparum* co-chaperone 'PFA0660w' complexes with major virulence factor 'PfEMP1' via chaperone 'PfHsp70-x'

Ankita Behl[1], Vikash Kumar[2], Anjali Bisht[3], Jiban J. Panda[3], Rachna Hora[2], Prakash Chandra Mishra[1]*

[1] Department of Biotechnology, Guru Nanak Dev University, Amritsar, Punjab, India

[2] Department of Molecular Biology and Biochemistry, Guru Nanak Dev University, Amritsar, Punjab, India

[3] Institute of Nano Science and Technology, Mohali, India

*Corresponding author name: Prakash Chandra Mishra; Email: mypcm@yahoo.co.in and Phone: 91 183 2258802-09 Extn: 3224

Supplementary material 1: *Western blot analysis using anti-hexahistidine and anti-PFA0660w antibodies.*

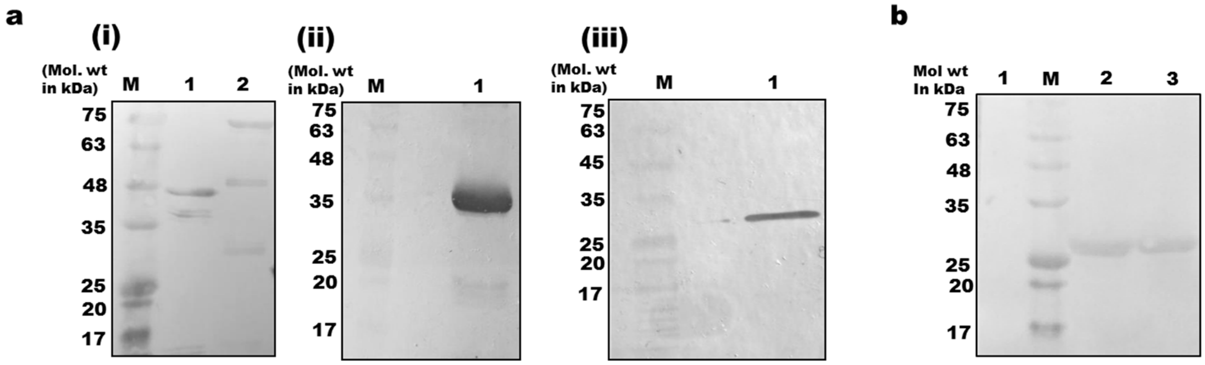

Figure S1. *Immunoblots showing identity of recombinant proteins (A) and specificity of polyclonal rabbit antibodies raised against PFA0660w-S (B).* **(a)** Detection of purified proteins using anti-hexahistidine antibodies. (i) Lane M: molecular weight marker, Lane 1: PfHsp70-x-S, Lane 2: PfHsp70-x-C. (ii) Lane M: molecular weight marker, Lane 1: PFA0660w-C. (iii) Lane M: molecular weight marker, Lane 1: PFA0660w-S. (**b)** Blots was probed using anti-PFA0660w primary antibodies (1:5000) followed by anti-rabbit secondary antibodies (1:2000). Lane 1: crude extract of *E. coli* BL21 (DE3) cells (negative control), lane M: Molecular weight marker, lane 2: crude extract of *E. coli* BL21 (DE3) cells transformed with PFA0660w-S-pET28a(+) plasmid, lane 3: recombinant PFA0660w-S (positive control).

Supplementary material 2: *Chromatograms of standards used in size exclusion chromatography.*

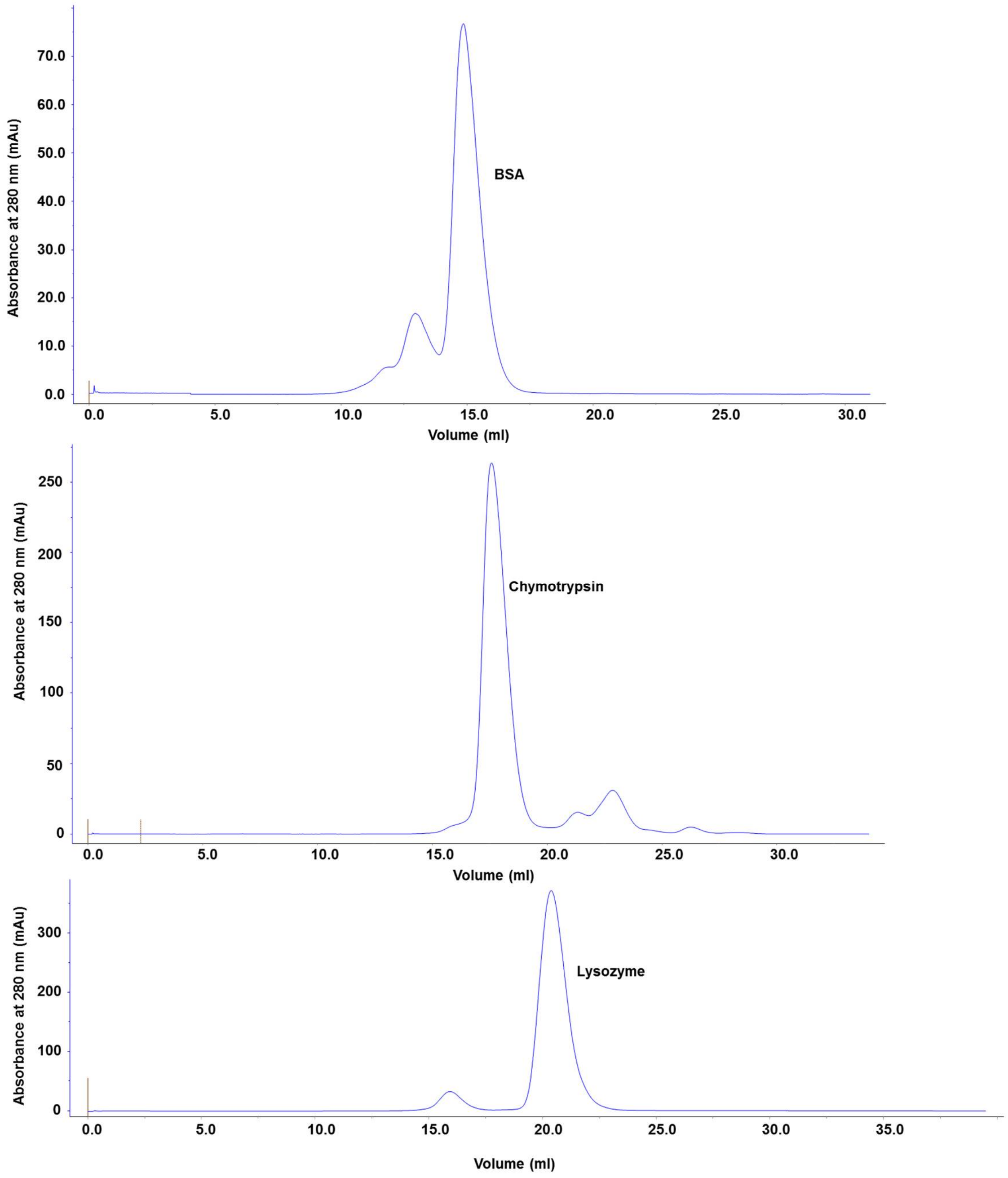

Figure S2. Standards (BSA, Chymotrypsin and lysozyme) were run on gel permeation column Superdex 200 10/300 GL. Labelled peaks indicate elution volume of respective proteins.

Supplementary material 3: *Ramachandran, Verify 3D and Errat plots validating generated models of J domain and C-terminal regions of PFA0660w.*

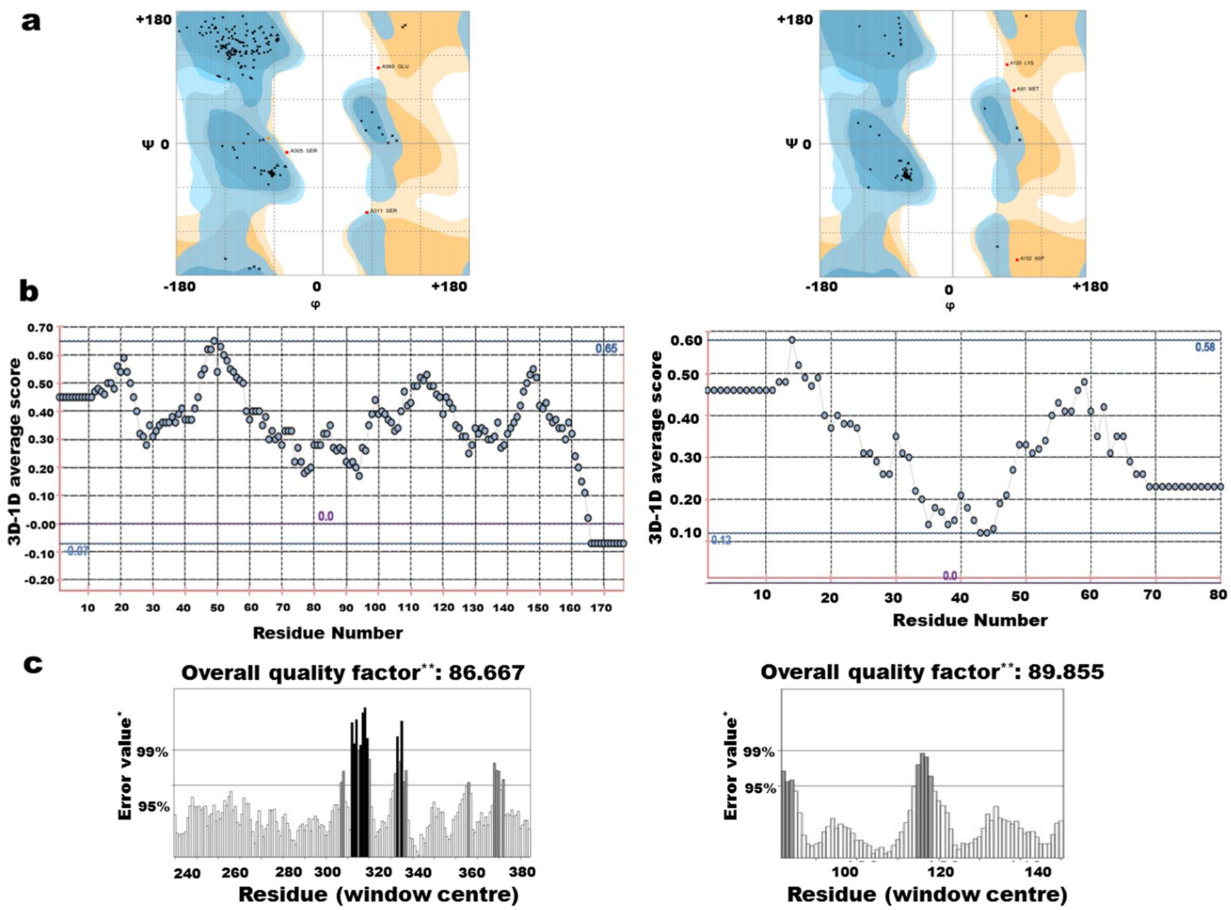

d

	J domain (78-154)	C terminal region (226-398)
**Rampage***	96%	97.7%
**Verify 3D**	100%	85.55%
**Errat**	89.85	86.66

* Percentage of residues in favoured region

Figure S3. Ramachandran (a), Verify 3D (b), and Errat plots (c) of modelled J domain (left panels) and C-terminal region (right panels) of PFA0660w. (d) Scores obtained for each of these plots are tabulated.

Supplementary material 4: *Multiple sequence alignment of Hsp40 family members from different species.*

a

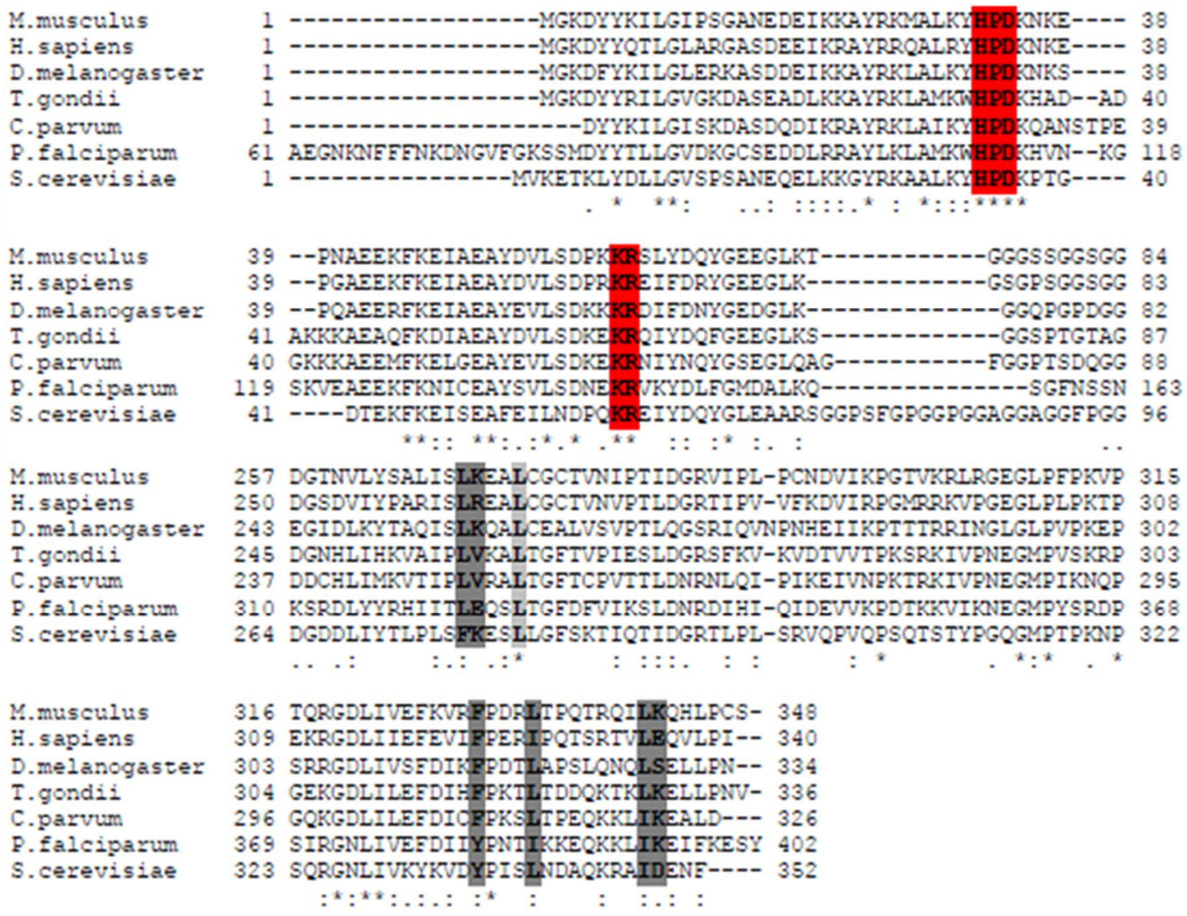

b

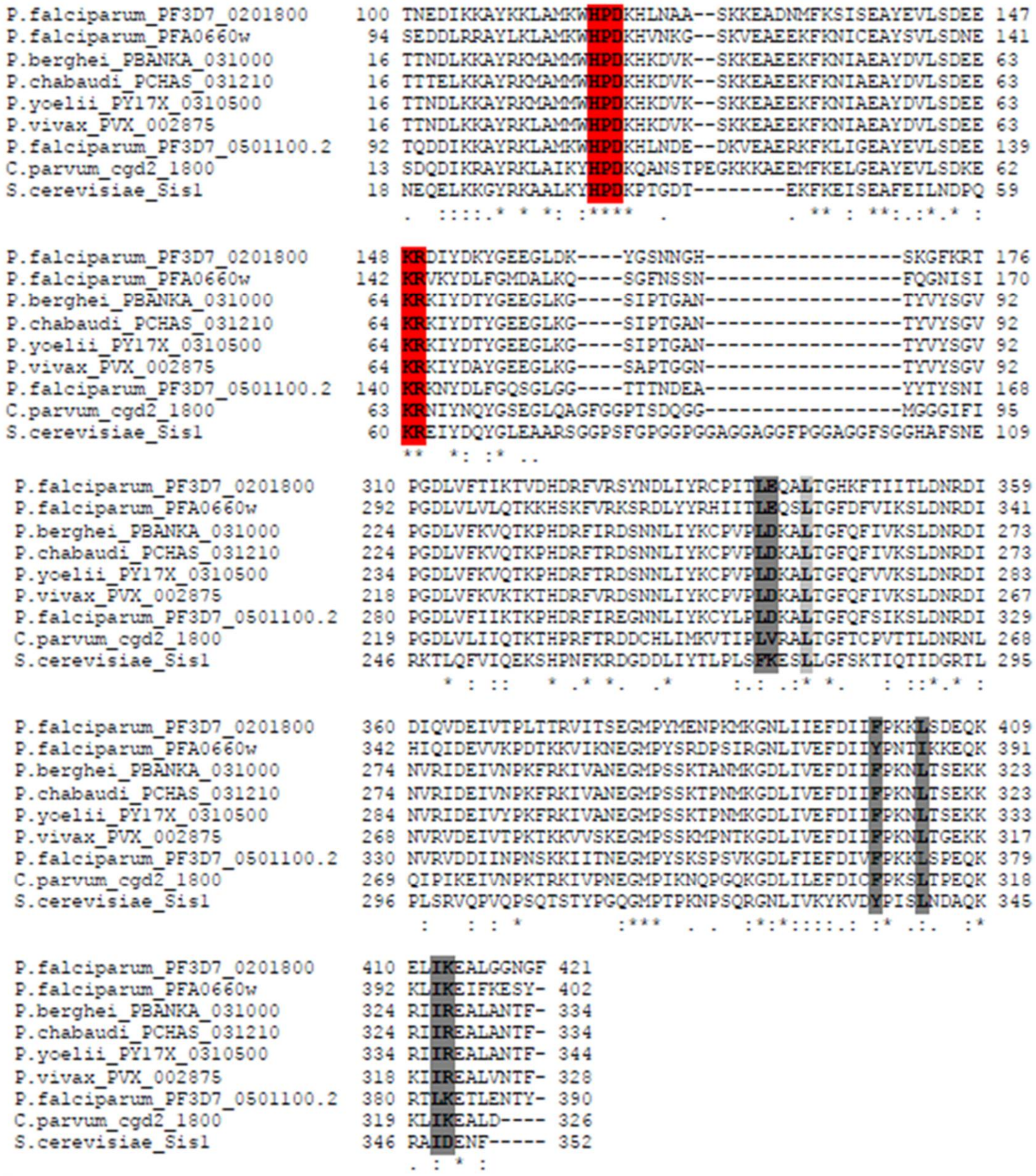

Figure S4. *Multiple sequence alignment of Hsp40 family members.* Residues known to be involved in Hsp70 interaction site in *E.coli* DnaJ and dimer interface of yeast Sis1 and their corresponding residues in other family members are shaded in red and grey respectively. **(a)** Multiple sequence alignment of PFA0660w with Hsp40 proteins from *M. musculus* (Hsp40-3), *H. sapiens* (Hdj1), *D. melanogaster* (Droj-1), *T. gondii* (TgSis1), *S. cerevisiae* (Sis1) and *C. parvum* (Cgd2_1800) using Clustal omega. **(b)** Multiple sequence alignment of Hsp40 homologs of PFA0660w in *P. falciparum 3D7* (PF3D7_0201800, PF3D7_0501100.2) and other *Plasmodium* species including *P. vivax* (PVX_002875), *P. berghei* (PBANKA_0310000), *P. chabaudi* (PCHAS_031210) and *P. yeolii* (PY17X_0310500).

Supplementary material 5: *Blots testing cross-reactivity of different antibodies against various proteins/lipids used in the study and raw data for MDH aggregation suppression assay.*

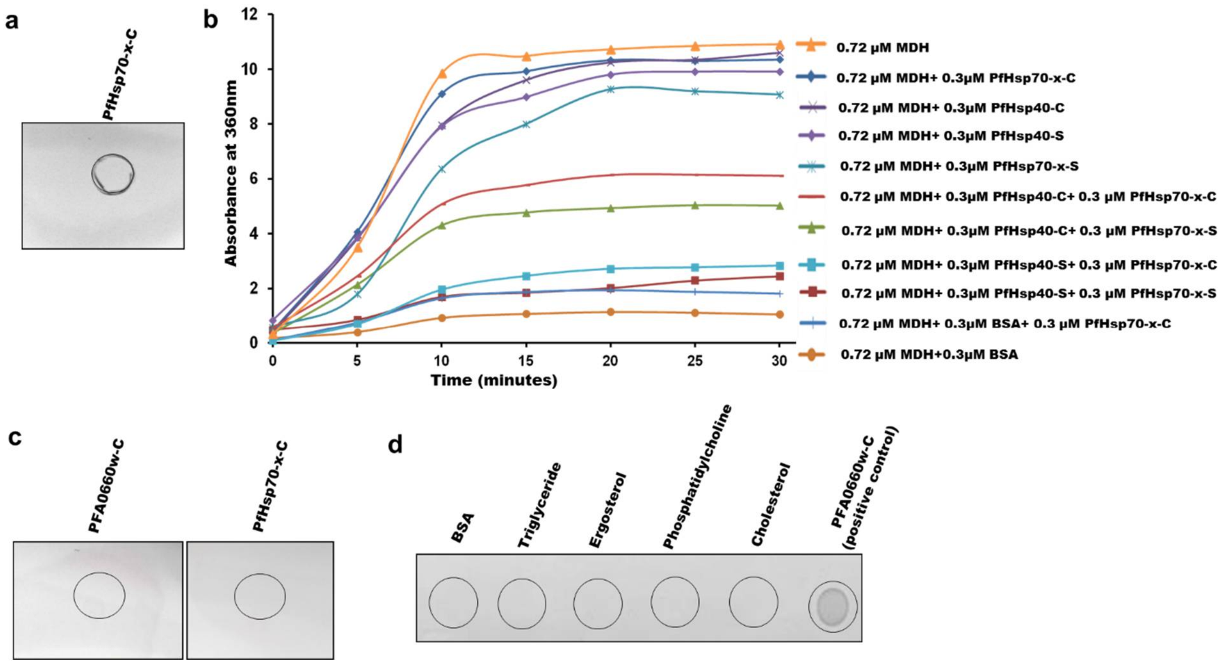

Figure S5. a) PfHsp70-x was spotted, and probed using anti-PFA0660w-S antibodies (1:5000) before incubating with goat anti-rabbit HRPO (1:2000) as secondary antibodies. **b)** *Raw data for MDH aggregation suppression assay.* MDH aggregation was monitored spectrophotometrically at 360nm, and considered 100% at saturation (average of absorbance values at 20, 25, 30 minutes) for MDH only vial. Results in figure 4a were plotted as bar diagram by taking an average of these three time points for samples corresponding to MDH-chaperone/control combinations. **c, d)** Respective proteins/lipids were spotted, and probed using c) anti-ATS (1:5000) and d) anti-hexahistidine-HRP (1:2000) as primary antibodies. Goat anti-mice HRPO (1:2000) (c) were used as secondary antibodies. The spotted proteins/lipids are marked on the respective blots.

__Supplementary material 6:__ Complete blots/gels corresponding to figures 2, 3, 5 and 7.

Figure 2:

a

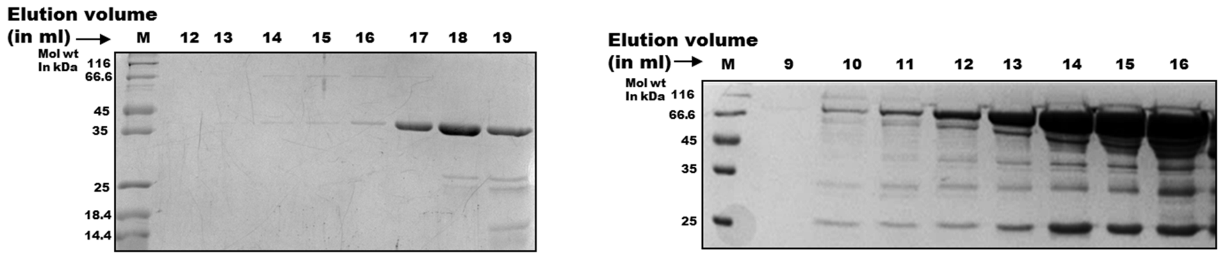

Figure 3:

d

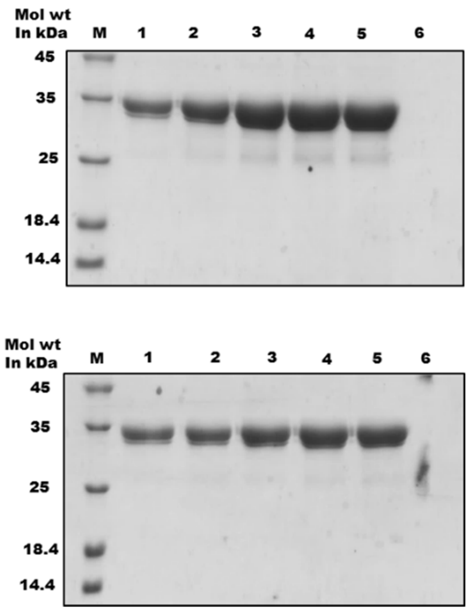

Figure 5:

c

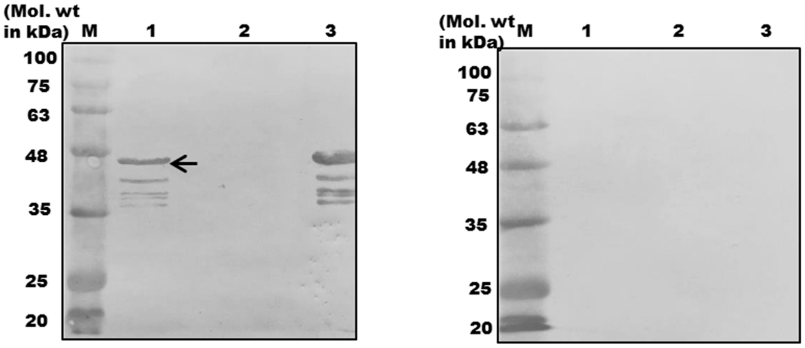

Figure 7:

b

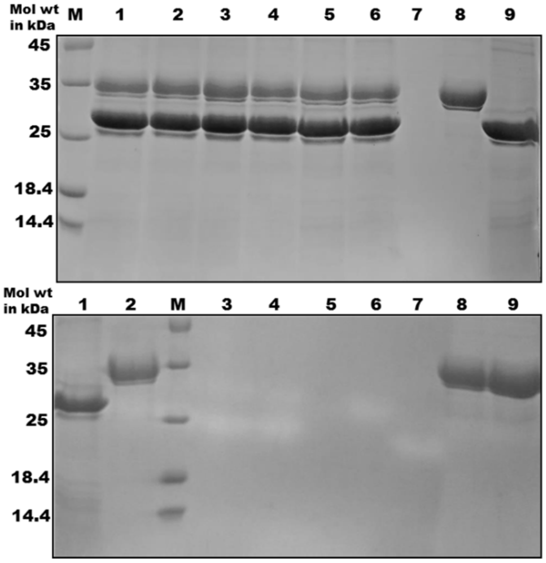

c

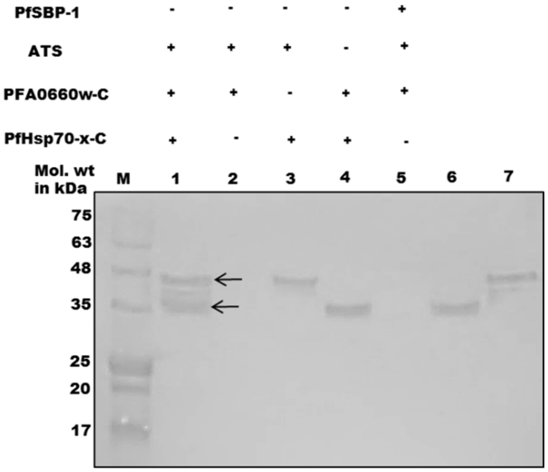

Table S1: List of predicted interactions in *C. parvum* Hsp40 (PDB ID: 2Q2G) and *S. cerevisiae* Sis1 (PDB ID: 1C3G) as computed by Protein Interaction Calculator server. Polypeptide chains of atomic resolution structures of the corresponding residues are shown in parenthesis.

	C. parvum Hsp40 **(PDB ID:2Q2G)**	***S. cerevisiae*** **Sis1 (PDB ID: 1C3G)**
**Hydrophobic Interactions**	249 L(A)-249 L(B), 249 L (A)-253 L(B), 249 L(A)-309 F(B), 249 L (A)-325 L(B), 250 V (A)-321 L(B), 250 V (A)-325 L(B), 252 A (A)-309 F(B), 253 L (A)-249 L(B), 253 L (A)-309 F(B), 253 L (A)-310 P(B), 253 L (A)-313 L(B), 253 L (A)-321 I(B), 253 L (A)-325 L(B), 275 I (A)-309 F(B), 275 I (A)-310 P (B), 275 I (A)-313 L(B), 278 P(A)-278 P(B), 278 P(A)-309 F (B), 307 I(A)-309 F(B), 309 F(A)-249 L(B), 309 F(A)-252 A(B), 309 F(A)-253 L(B), 309 F(A)-275 I (B), 309 F(A)-278 P(B), 309 F(A)-307 I(B), 309 F(A)-309 F(B), 310 P(A)-253 L(B), 310 P(A)-275 I(B), 310 P(A)-325 L(B), 313 L(A)-253 L(B), 313 L(A)-275 I(B), 313 L(A)-325 L(B), 320 L(A)-324 A(B), 321 I(A)-250 V(B), 321 L(A)-253 L(B), 321 L(A)-324 A(B), 321 L(A)-325 L(B), 321 L(A)-320 L(B), 324 L(A)-321 I(B), 325 L(A)-249 L(B), 325 L(A)-250 V(B), 325 L(A)-253 L(B), 325 L(A)-310 P(B), 325 L(A)-313 L(B), 325 L(A)-321 I(B)	276 F(A)-276 F(B), 276 F(A)-280 L(B), 276 F(A)-336 Y(B), 276 F(A)-348 I(B), 280 L(A)-276 F(B), 280 L(A)-336 Y(B), 280 L(A)-337 P(B), 280 L(A)-340 L(B), 280 L(A)-348 I(B), 281 L(A)-340 L(B), 281 L(A)-348 I(B), 302 P(A)-336 Y(B), 302 P(A)-337 P(B),302 P(A)-340 L(B), 305 P(A)-305 P(B), 305 P(A)-334 V(B), 305 P(A)-336 Y(B), 334 V(A)-305 P(B), 334 V(A)-336 Y(B), 336 Y(A)-276 F(B), 336 Y(A)-280 L(B), 336 Y(A)-302 P(B), 336 Y(A)-305 P(B), 336 Y(A)-334 V(B), 336 Y(A)-336 Y(B), 337 Y(A)-280 L(B), 340 L(A)-280 L(B), 340 L(A)-281 L(B), 340 L(A)-302 P(B), 347 A(A)-347 A(B), 348 I(A)-276 F(B), 348 I(A)-280 L(B), 348 I(A)-281 L(B)
**Hydrogen bonds**	317 Q(A)-324 A(B), 318 Q(A)-273 K(B), 324 A(A), 318 Q(B)	304 Q(A)-336 Y(B), 279 S(A)-336 Y(B), 339 S(A)-301 Q(B)
**Ionic interactions**	279 K(A)-306 D(B), 318 K(A)-274 Q(B)	349 D(A)-277 K(B)
